# Antibiogram profiling and detection of *icaA* and *blaZ* genes from *Staphylococcus aureus* and coagulase-negative *Staphylococcus* spp. of healthy bovine raw milk sample origin

**DOI:** 10.5455/javar.2024.k795

**Published:** 2024-06-19

**Authors:** Asmaul Husna, Md. Arefin Kallol, Farhana Binte Ferdous, Khudaza Akter Lima, Zannatul Haque Tumpa, Mohammad Ferdousur Rahman Khan, Marzia Rahman

**Affiliations:** Department of Microbiology and Hygiene, Bangladesh Agricultural University, Mymensingh Bangladesh

**Keywords:** *blaZ*, CoNS, *icaA*, MDR, Biofilm, *S. aureus*

## Abstract

**Objective:**

This study focused on the antibiogram profiling of *Staphylococcus aureus *and coagulase-negative *Staphylococcus *spp*.* (CoNS) and the detection of *icaA* and *blaZ *genes from bovine raw milk samples.

**Materials and Methods:**

Bovine milk samples were collected from dairy farms, and *Staphylococcus *spp. were isolated and identified via conventional and molecular screening. Disk diffusion test (DDT) was implemented to determine the resistance pattern. Biofilm and β-lactamase-producing *Staphylococcus* spp. were identified via amplification of the *icaA* and* blaZ* genes. Methicillin-resistant *Staphylococcus aureus* and CoNS were identified by DDT and PCR of the *mecA* gene.

**Results:**

From 63 samples, 35 were confirmed as *Staphylococcus *spp., of which 16 (25.39%) *S. aureus* isolates were coagulase-positive, while 19 (30.16%) were negative. PCR confirmed that 50% (8/16) of *S. aureus* and 36.84% (7/19) of CoNS possessed the *icaA *gene. All *S. aureus* isolates were found resistant to penicillin-G (P) both phenotypically and genotypically. The isolates were also resistant to erythromycin (ERY) and oxytetracycline (TET). While CoNS showed high to reduced resistance against P, TET, ERY, and azithromycin, no *S. aureus* isolates were resistant to sulfamethoxazole, while 10.53% of CoNS isolates were. All *S. aureus *and CoNS isolates were susceptible to vancomycin and gentamicin. MR was exhibited by 37.5% of *S. aureus* and 42.10% of CoNS isolates. Moreover, *S. aureus* and CoNS had 56.25% and 52.63% multidrug-resistant (MDR) isolates, respectively.

**Conclusion:**

The present study revealed the presence of a biofilm-producing, MDR staphylococcal strain in milk that might endanger consumers. Routine surveillance and monitoring, along with antimicrobial resistance learning, can reduce risks.

## Introduction

Most mastitis is caused by bacteria; however, viral, algal, and fungal strains have also been reported. The teat canal accommodates bacteria into the bovine mammary gland, causing “mastitis” [1]. Nearly 200 infectious agents of mastitis in bovine have been identified till now, with Coliforms, *Streptococcus agalactiae*, *Staphylococcus aureus*, and other *Streptococci* being the most prevalent microbes found in large animals [2]. *S. aureus* is the primary cause of bovine mastitis, the most frequent dairy complication [3]. Although mastitis among cattle is induced by plenty of factors, *S. aureus* is the primary global cause of bovine mastitis [4]. *S. aureus*, a major food-borne pathogen, infects humans and animals [5]. Recently, in most countries, coagulase-negative staphylococci (CoNS) elicit intramammary infections (IMIs), despite *S. aureus* being the most dangerous mastitis-causing pathogen in cattle [6].

Nevertheless, clinical or pathogenic significance while isolated from milk remains controversial; some address mastitis-causing bacteria with significant virulence factors [7]. The key pathogenic hallmark of *S. aureus* and CoNS in mastitis is biofilm formation. Researchers recently discovered approximately 15 CoNS species that induce IMI in dairy cattle, but *Staphylococcus epidermidis*, *Staphylococcus haemolyticus*, *Staphylococcus simulans*, *Staphylococcus hyicus*, *Staphylococcus chromogenes, *and *Staphylococcus xylosus* are the predominantly isolated CoNS from mastitis in dairy cattle [8]. Various species have been recorded. Due to the rising role of CoNS in bovine mastitis, species-level CoNS detection is crucial to developing efficient management approaches [9].

Clusters of microorganisms on various surfaces are called “biofilm.” Biofilm formation in *S. aureus *boosts the expansion of antibiotic resistance. Many genes contribute to biofilm formation; however, the *icaA* gene is a crucial factor for *S. aureus* strains. The *icaA* gene produces a transmembrane protein that synthesizes poly-N-acetylglucosamine polymer via N-acetylglucosaminyl transferases [10].

Antibiotic-resistant mastitis-causing bacteria have been reported to result from antibiotic abuse. The diverse therapeutic use of antimicrobials or their frequent use as growth stimulants in animal feed has been associated with human and animal-borne pathogenic microorganism resistance [11]. Nowadays, bovine mastitis treatment begins with antibiotics, and the resultants’ resistant microbes are rendering antibiotics ineffective. Antibiotic residues additionally jeopardize public health [12]. The detection of pathogens in mastitis is crucial for antibiotic selection. β-lactams are extensively utilized in intramammary medication. Anti-β-lactam strategies in bacteria include *blaZ* gene-encoded β-lactamases and *mecA* gene-encoded low affinity penicillin-binding protein (PBP2a). The methicillin-resistance (MR) condition prohibits treatment with known β-lactam antibiotics [13]. It is predicted that the occurrence of MR in the CoNS is greater than that of *S. aureus*. The *mecA *gene is harbored by MR CoNS, which can be horizontally transferred among staphylococci. Moreover, *mecA-*positive CoNS might act as vectors for spreading newly detected clones of Methicillin-resistant *Staphylococcus aureus* (MRSA) [14]. In Bangladesh, there has been no published research on biofilm-producing CoNS detection in mastitis-infected cows. The current study assessed antibiotic-resistant and *icaA* genes containing *S. aureus* and *S. epidermidis* in raw milk from selected farms.

## Materials and Methods

### Sample collection

From the dairy farms of Bangladesh Agricultural University (BAU) (24.7363°N, 90.4245°E) and Nitish Bohumukhi Dairy Farm, Pulbaria, Mymensingh (24.57961°N, 90.0770°E), a total of 63 fresh milk samples (32 from the BAU dairy farm and 31 from Fulbaria) were collected from wholesome lactating cows utilizing sterilized apparatus. About 10 ml of milk was drawn at random from a single quarter. The milk samples were collected without harming the cows following the guidelines set by the ethical committee of the BAU. The research was conducted at the laboratories of the Department of Microbiology and Hygiene, BAU, Mymensingh.

### Isolation of Staphylococcus spp.

Test specimens (0.01 ml, milk) had been streaked over 5% sheep blood agar and incubated overnight at 37°C suspected *Staphylococcus* spp. colonies were sub-cultured on MSA for pure culture. Colony characteristics on MSA, β-hemolytic motifs on blood agar supplemented with sheep blood (5%, v/v), Gram staining properties, catalase, and coagulase assays confirmed the isolates as *Staphylococcus *spp. Fresh rabbit plasma and 3% hydrogen peroxide were utilized for catalase and coagulase tests. Finally, *Staphylococcus* spp. was verified by amplification of the *nuc *gene.

### Extraction of bacterial genomic DNA

In a nutshell, for the extraction, simply one colony had to be placed in the distilled water of 100 µl in an Eppendorf tube, thoroughly mixed, and heated for around 10 min. After heating, tubes were placed on ice for cold shock and centrifuged at 10,000 rpm for 10 min at 4°C. The supernatant was separated and used as a DNA template [15].

### Amplification of nuc, mecA, icaA, and blaZ genes by PCR

Using a gradient-based thermocycler, simplex PCR was carried out to amplify the *nuc* and *mecA *genes and identify MRSA. Genes associated with biofilm (*icaA*) and β-lactamase production (*blaZ*) had been amplified individually. Genomic DNA that had tested positive previously for the specified genes was utilized as positive controls. Non-template controls were established using PBS instead of genomic DNA for negative controls. The thermal profiles of three PCRs *(*first PCR: *nuc* plus *mecA*; second PCR:* icaA*; third PCR: *blaZ*) were initial denaturation at 95°C for 5 min; the sample underwent 30 cycles of denaturation at the same temperature for 1 min; final extension at 72°C for 10 min; and holding at 4°C. The annealing temperatures were 55°C for 45 sec, 50°C for 30 sec, and 46°C for 30 sec to amplify the genes (*nuc* plus *mecA, icaA,* and *blaZ*), respectively. The extension temperatures of these three PCR’s were 72°C for 45 sec, 72°C for 1.5 min, and 72°C for 45 sec. The detailed information on primers is presented in Table 1.

### Antimicrobial susceptibility testing

Kirby-Bauer’s disc diffusion technique (DDT) [18] was applied to determine antimicrobial susceptibility. The results were presented according to CLSI standards [19]. Ten antibiotic discs from seven different classes were used for the study. By adding normal saline, each isolate’s overnight growth was set to a concentration of 0.5 McFarland. Bacterial cultures were dispersed on Muller-Hinton agar with sterile cotton buds and then air-dried. The antibiotic discs of each group were placed on the bacterial lawn, which was incubated overnight at 37°C, followed by a recording of the zone of inhibition and analyses of the findings. Finally, findings have been described as susceptible (S) and resistant (R). Ferdous et al. [18] described multidrug-resistant (MDR) isolates as those that resist at least one compound from each of the three antimicrobial classes.

### Statistical analysis

The Statistical Package for Social Science (SPSS.v.25, IBM, Chicago, IL) was used to conduct the statistical tests. Through descriptive analysis, the prevalence of various variables was calculated. The *chi-*square test for relatedness was conducted to find out whether or not the frequencies of resistance genes differed. Additionally, an identical test was done to check the variations in the occurrence of phenotypic antibiotic resistance in relation to the presence of resistance genes.

## Results 

### Occurrence of Staphylococcus spp. in milk

Out of 63 samples, 35 (55.55%) isolates were suspected as *Staphylococcus* spp., of which 16 (6 from BAU and 10 from Fulbaria) and 19 (7 from BAU and 12 from Fulbaria) isolates ensured their identities as *S. aureus* CoNS, respectively. In this study, the rates of coagulase-positive *Staphylococcus* and CoNS in milk were 25.39% (16/63) and 30.15% (19/63), respectively.

### Detection of biofilm-producing genes

**Figure 1.**
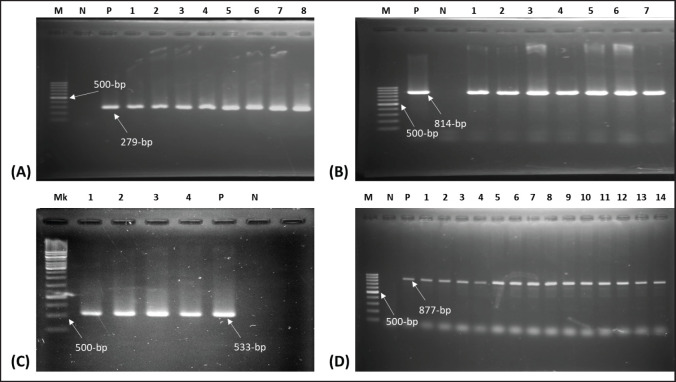
Amplification of genes in *Staphylococcus* spp. (A) *nuc* gene (279–bp) of *S. aureus*. Lane 1–8: test samples, (B) *icaA gene* (814–bp) gene of *Staphylococcus *spp. Lane 1–7: test samples, (C) *mecA* gene (533–bp) of *Staphylococcus *spp., Lane 1–4: test samples, (D) *blaZ gene *(877–bp) of *Staphylococcus *spp. Lane 1–14: test samples M: 100-bp DNA ladder, Mk: 1kb ladder, P: positive control, N: negative control.

Biofilm producing the *icaA* gene was detected in both coagulase-positive and CoNS samples by PCR. Out of 35 samples, the overall occurrence of the *icaA* gene was detected in 42.86% (15/35) isolates, of which 50% *S. aureus* and 36.84% CoNS isolates were found to be harboring the *icaA* gene, respectively (Table 2; Fig. 1B).

### Antibiogram of Staphylococcus spp

**Figure 2.**
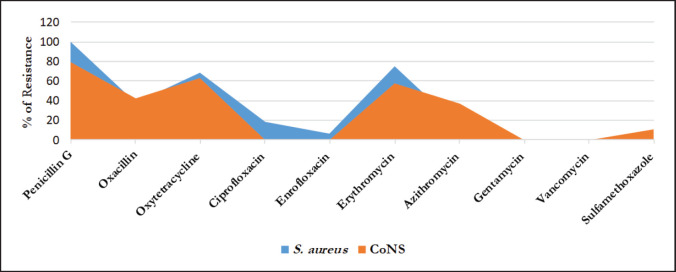
Antimicrobial resistance patterns of *Staphylococcus aureus* and coagulase negative *Staphylococcus* spp. (CoNS).

Figure 2 depicts the resistance pattern found in both *S. aureus* and CoNS. The pattern of antibiotic resistance showed that all the isolates of *S. aureus* (100%) were found resistant to penicillin-G, 75% to erythromycin (ERY), 68.75% to oxytetracycline (TET), and 37.5% to methicillin. On the other hand, 73.68% of CoNS were resistant to penicillin-G, 63.16% to TET, 57.89% to ERY, 42.10% to methicillin, and 36.84% to azithromycin (AZM). *S. aureus* isolates had all been sensitive to sulfamethoxazole (SUL), gentamicin, and vancomycin, while all CoNS isolates were responsive to SUL and gentamicin.

### Relationship of phenotypic and genotypic antibiotic resistance patterns with biofilm-producing genes

**Figure 3.**
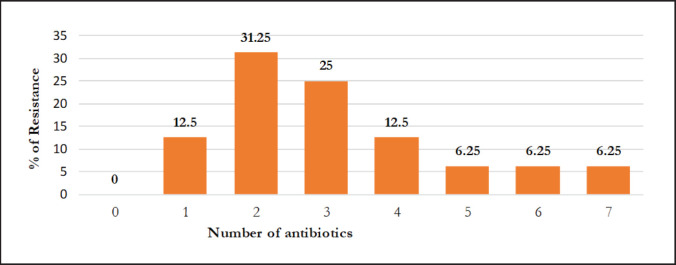
Resistance pattern of *Staphylococcus aureus.*

**Figure 4.**
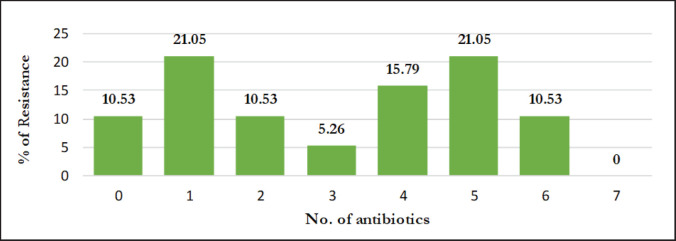
Resistance pattern of coagulase-negative *Staphylococcus *spp*.* (CoNS).

Among 16 isolates of *S. aureus*, 50% were noticed to be *icaA*-bearing, of which 6 (37.75%) were phenotypically and genotypically resistant to oxacillin (OXA). Statistical analysis showed that OXA-resistant isolates carried significantly higher amounts of the* mecA* gene than the *icaA* and *blaZ* genes (Table 5). Furthermore, 7 (43.75%) *mecA*-positive isolates (Fig. 1C) indicate MR, and all are *blaZ*-positive (Fig. 1D) with resistance to penicillin-G, which are presented in Table 4. However, among the 19 isolates of CoNS, 7 isolates (36.84%) were detected as *icaA-*bearing*,* of which all were positive for *blaZ* (Table 6). Although the total *blaZ-*positive CoNS isolates were 78.95%, the results revealed that 56.25% and 52.63% isolates of *S. aureus* and CoNS were recognized as MDR because these isolates exhibited resistance to antibiotics from three or more distinct classes (Table 3). Maximum 5 (31.25%) isolates of *S. aureus* showed resistance to two antibiotics, and 6.25% isolates showed resistance to 5, 6, and 7 antibiotics, respectively. In the case of CoNS, a maximum of 21.05% of isolates were observed to be resistant to 5 antibiotics (Figs. 3 and 4). For *S*.* aureus,* the multiple antibiotic resistance (MAR) indices varied from 0.1 to 0.7, while for CoNS, they were 0.1–0.6.

**Table 1.** Primers used in this study.

Name of Primers	Targeted Gene	Primer’s Sequences (5′-3′)	Amplicon size (bp)	References
*nuc F*	*nuc*	GCGATTGATGGTGATACGGT	279	[16]
*nuc R*		AGCCAAGCCTTGACGAACTAAAGC		
*ica*A* F*	*icaA*	GACCTCGAAGTCAATAGAGGT	814	
*ica*A* R*		CCCAGTATAACGTTGGATACC		
*mec*A* F*	*mecA*	AAAATCGATGGTAAAGGTTGG	533	[17]
*mec*A* R*		AGTTCTGGCACTACCGGATTTTGC		
*bla*Z* F*	*blaZ*	TCAAACAGTTCACATGCC	877	
*bla*Z* R*		TTCATTACACTCTGGCG		

**Table 2.** Occurrence rate of *icaA* bearing *S. aureus* and CoNS isolated from milk samples.

Sample type and no.	Isolated organisms	Positive sample	Occurrence rate in %	No of *icaA* positive isolates	% of *icaA* bearing isolates
63 milk	*S. aureus*	16	25.39	8	50
	CoNS	19	30.16	7	36.84
Total		35	49.21	15	42.86

## Discussion

*Staphylococcus *spp., a prevalent zoonotic microorganism of dietary origin, causes many human and livestock illnesses. Colonization in the mammary glands of dairy cattle causes mastitis and other syndromes that contaminate raw milk. Ingestion of raw or inadequately boiled milk can cause a staphylococcal infection. *Staphylococcus aureus* and CoNS are the leading mastitis pathogens and biofilm producers that concern public health [20].

**Table 3.** Multidrug resistance pattern of *S. aureus* and CoNS.

*Staphylococcus aureus*				Coagulase-negative Staphylococcus *spp*. (CoNS)			
Sample ID	Phenotypic resistance profile	Resistance type	MAR index	Sample ID	Phenotypic resistance profile	Resistance type	MAR index
BAU 1	P, TET	-	0.2	BAU 1	P, ERY		0.2
BAU-3	P, OXA, TET, CIP, ERY	MDR	0.5	BAU-3	P, OXA, TET, ERY, CN	MDR	0.5
BAU-7	P, ERY		0.2	BAU-4	P		0.1
BAU-14	P, OXA, TET, CIP, EN, ERY, CN	MDR	0.7	BAU-7	P, OXA, TET, ERY, CN, SUL	MDR	0.6
BAU-19	P, OXA, TET, ERY	MDR	0.4	BAU-11	P		0.1
BAU-24	P, ERY, TET	MDR	0.3	BAU-19	P, TET, ERY, CN	MDR	0.4
BAU-25	P	-	0.1	BAU-20	P, OXA, TET, ERY, CN	MDR	0.5
F-4	P, OXA, TET, ERY	MDR	0.4	BAU-25	P		0.1
F-9	P, ERY, TET	MDR	0.3	F-2	P, OXA, TET, ERY, CN	MDR	0.5
F-13	P		0.1	F-9	P, TET		0.2
F-14	P, ERY, TET	MDR	0.3	F-12	P, TET, ERY	MDR	0.3
F-18	P, ERY	-	0.2	F-14	P, TET	-	0.2
F-27	P, ERY	-	0.2	F-20	-	-	-
F-33	P, OXA, TET, ERY, CIP, CN	MDR	0.6	F-21	-	-	-
F-34	P, TET	-	0.2	F-22	P, OXA, TET, ERY, CN, SUL	MDR	0.6
F-37	P, OXA, TET, ERY	MDR	0.4	F-33	P, OXA, TET, ERY	MDR	0.4
% of MDR = 56.25				F-34	P, OXA, TET, ERY	MDR	0.4
F-35	P, OXA, TET, ERY, CN	MDR	0.5
F-38	P		0.1
% of MDR = 52.63			

**Table 4.** Occurrence of *blaZ, mecA* and *icaA* genes in isolated *Staphylococcus *spp.

Type of *Staphylococcus*	No. of isolates	% of *blaZ* gene	% of *mecA* gene	% of *icaA* gene	*p*-value
*S. aureus*	16	100 (16/16)	37.5 (6/16)	50 (8/16)	0.013
CoNS	19	78.95 (15/19)	42.10 (8/19)	36.84 (7/19)	0.002

Variables differ significantly at *p *< 0.05 level.

**Table 5.** Association of phenotypic and genotypic resistance pattern in *S. aureus.*

**Resistanc**e	**Genotypic**				
	Antibiotics	No (%) of *blaZ* (*n = *16)	No (%) of *mecA* (*n = *6)	No (%) of *icaA* (*n = *8)	*p*-value
Phenotypic	P	16 (100^a^)	6 (100^a^)	8 (100^a^)	NA
	OXA	6 (37.5^a^)	6 (100^b^)	6 (75^a^)	0.017
	TET	11 (68.8^a^)	6 (100^a^)	8 (100^a^)	0.072
	CIP	3 (18.8^a^)	3 (50^a^)	3 (37.5^a^)	0.313
	ERY	12 (75^a^)	6 (100^a^)	7 (87.5^a^)	0.350
	EN	1 (6.3^a^)	1 (16.7^a^)	1 (12.5^a^)	0.508
	CN	2 (12.5^a^)	2 (33.3^a^)	2 (25^a^)	0.740

Here, values with different superscripts differ significantly (*p *< 0.05) within the variable under assessment, NA = Not applied.

**Table 6.** Association of phenotypic and genotypic resistance pattern in CoNS.

Resistance	Genotypic
Antibiotics	No (%) of *blaZ* (*n = *15)	No (%) of *mecA* (*n = *8)	No (%) of *icaA* (*n = *7)	*p*-value
Phenotypic	P	14 (93.3^a^)	8 (100^a^)	7 (100^a^)	0.596
	OXA	8 (53.3^a^)	6 (75^b^)	6 (85.7^a^)	0.274
	TET	12 (80^a^)	8 (100^a^)	7 (100^a^)	0.189
	ERY	11 (73.3^a^)	7 (87.5^a^)	6 (85.7^a^)	0.657
	CN	7 (46.7^a^)	7 (87.5^a^)	6 (85.7^a^)	0.067
	SUL	2 (13.3^a^)	2 (25^a^)	2 (28.6^a^)	0.650

Here, values with different superscripts differ significantly (*p* < 0.05) within the variable under assessment, NA = Not applied.

Although Ballah et al. [16] reported the morphological and genotypic features of biofilm-forming *S. aureus* from Bangladesh for the first time, The continuing study prioritized CoNS and *S. aureus* to determine their association or differentiation based on phenotypic resistance patterns and the presence or absence of *blaZ*, *mecA, *and *icaA* genes, and noticed* S. aureus* and CNS at rates of 25.39% and 30.16%, respectively. Previously, Jahan et al. [21] isolated *S. aureus* from milk, similar to this study but greater than Dai et al. [3] and lower than Atyabi et al. [22] and Hashemi et al. [23]. Atyabi et al. [22] found 30.27% and 2.89% prevalence in CoNS and *S. aureus,* respectively. Whereas, it was revealed that 19.56% of milk was coagulase-positive and 12.53% had CoNS, concerning staphylococci as the most predominant bacteria [22,23]. André et al. [24] reported 73% *S. aureus* at a dairy manufacturing facility in Goiás, Brazil, among which 75%, 70%, and 66.7% were detected from milk handlers, cheese, and milk samples, respectively.

The study uncovered that milk handlers, milking equipment, and mammary glands of healthy dairy cattle may contaminate milk with CoNS and *S. aureus *causing clinical and subclinical mastitis. Konuku et al. [6] stated that an elevated rate of *S. aureus* indicates poor milking, transportation, and dissemination. The appropriate heating process before refrigeration may reduce *S. aureus* risk. Immunocompromised individuals, infants, the elderly, and women with pregnancies are most vulnerable to raw milk’s bacteria. The pathogenicity of the *Staphylococcus* genus is regulated by the *ica* operon, which encodes the polysaccharide intercellular adhesin (PIA) [25]. Using PCR, this study found the *icaA* gene in *S. aureus* and CoNS at 50% and 36.84%, respectively, which was lower than Gajewska et al. [26]. In those findings Gajewska et al. [26] noted the *icaA* gene only in CoNS (24.1%) and no *icaA* gene in *S. aureus*; however, the current research has detected it in both coagulase-positive and negative strains. A mutation on *icaA* may cause DNA sequence changes that prevent these genes from being amplified [27].

Khairullah et al. [28] isolated 5.15% and 4.22% of MDR *S. aureus* and CoNS isolates from cow milk and farmers’ hands in East Java, Indonesia, which was significantly lower than the present study. Patterns in healthcare infrastructure, regulatory policies, socioeconomic conditions, climate, and geography all contribute to this change. The isolates harboring *icaA* gene showed multidrug resistance as well as MR, which might indicate a strong biofilm producer. Intrinsically, the biofilm-producing genes stimulate bacteria to express themselves as strong biofilm producers, leading to the development of the MDR strain. Phenotypic biofilm production is extremely controlled by some genes; among these, *icaA *is the most common gene. The presence of a higher percentage of *icaA* bearing MDR *S. aureus* and CoNS in milk might pose a risk to human and animal health. Further investigation can be conducted on the biofilm assay using CRA (Congo Red Agar) and the microtiter plate method, along with molecular detection of other biofilm-forming genes.

## Conclusion

Though ubiquitous, staphylococci can cause subclinical and persistent intra-mammary infections in cows through various virulence factors. We tested bovine milk from selected farms for *Staphylococcus* spp. Phenotypic and molecular characteristics identified the isolates as *Staphylococcus *spp. In isolated strains, *S. epidermidis* was more common than *S. aureus*. Biofilm-forming probability and MR were genotypically checked by PCR. Antibiograms showed susceptibility to penicillin-G, TET, ERY, AZM, and OXA. In summary, *S. aureus* in raw milk indicates food-borne infections and antibiotic resistance. Regular and rigorous observation and hygiene may reduce the danger.

## List of abbreviations

AZM, azithromycin; CIP, Ciprofloxacin; EN, Enrofloxacin; ERY, erythromycin; GEN, gentamycin; ml, milliliter OXA, oxacillin; P, penicillin G; SUL, sulfamethoxazole;TET, oxytetracycline; VA, vancomycin.
